# How and when workplace ostracism influences employee deviant behavior: A self-determination theory perspective

**DOI:** 10.3389/fpsyg.2022.1002399

**Published:** 2022-10-18

**Authors:** Jian Luo, Shuang Li, Lizhu Gong, Xueying Zhang, Siwei Wang

**Affiliations:** ^1^School of Business Administration, Southwestern University of Finance and Economics, Chengdu, China; ^2^Department of Accounting, Sichuan Tianyi College, Deyang, China

**Keywords:** basic psychological needs, deviant behavior, perceived inclusive climate, workplace ostracism, self-determination theory (SDT)

## Abstract

Drawing on self-determination theory, this study examines the relationship between workplace ostracism and deviant behavior by focusing on the mediating role of basic psychological needs and the moderating role of perceived inclusive climate. Findings based on the analysis of 247 valid survey samples suggest that (1) workplace ostracism has a significant positive impact on employees’ deviant behavior; (2) basic psychological needs mediate the relationship between workplace ostracism and employees’ deviant behavior; and (3) employees’ perceived inclusive climate weakens the negative effect of workplace ostracism on basic psychological needs. This study develops new perspectives for workplace ostracism research, extends the factors that influence employees’ deviant behavior, and expands the boundary conditions of organizational difference in self-determination theory. Moreover, these empirical results provide important theoretical guidance to decrease employees’ deviant behavior in organizations.

## Introduction

Workplace ostracism, a kind of “cold violence,” is defined as employees perceiving that they are ignored or excluded by others in the work environment (Ferris et al., 2008). For instance, employees suffering from ostracism believe that their colleagues do not wish to speak with them and even avoid them during work. Ostracism is widespread in the organizational context; an investigation of Monster (a well-known recruitment website) showed that approximately 40% of interviewees had experienced silent treatment in the previous year (Liu and Zong, 2019). In another survey of more than 5,000 employees, 69% reported that they had suffered different degrees of workplace ostracism as a result of leaders’ and colleagues’ indifference or disregard (Hoel et al., 2017). In China, the differentiation climate and “circle” culture in organizations are relatively strong, so workplace ostracism is more common and persistent (Zhu et al., 2017; Deng et al., 2021). Therefore, it is particularly important to pay attention to the consequences of workplace ostracism in the Chinese context. Previous research has explored ostracism, focusing mainly on its negative effects on both individuals and organizations, such as increased stress (Deng et al., 2021), sabotage behaviors (Sarwar et al., 2020), turnover intention (Singh and Srivastava, 2021; Wang et al., 2021), emotional exhaustion (Anjum et al., 2022), knowledge hoarding (Bhatti et al., 2022), diminished organizational trust and social capital (Paşamehmetoğlu et al., 2022), organizational citizenship behavior (Hitlan et al., 2006; Choi, 2020), voice behavior (Li and Tian, 2016), and reduced job performance (Zhu et al., 2017; Al-Atwi et al., 2021).

Although researchers have explained the effects of workplace ostracism from different theoretical perspectives, the theories of conservation of resources (COR) and reciprocity are the most popular. COR theory states that people are always trying to maintain, protect, and construct resources that are important to them, and that the potential or actual loss of such resources will pose a threat (Hobfoll, 1989). Workplace ostracism is thought to lead to the loss of employee resources (Zhao and Xia, 2017; Sarwar et al., 2020). According to COR theory, this loss of resources affects the wellbeing, social relations, and work outcomes of individuals (De Clercq et al., 2019; Choi, 2020). The reciprocity principle is the core feature of social exchange theory, which emphasizes that individuals treat others in the same or equivalent way that they are treated (Blau, 1964). Thus, when employees realize that they are being ostracized by others, they take retaliatory actions such as deviant behavior (Shafique et al., 2021). In addition to productive errors, violating organizational norms, such as purposely arriving at the workplace late or leaving early and stealing public assets can also undermine the wellbeing of the organization and its members (Zappalà et al., 2022). There are only a few studies about the relationship between workplace ostracism and deviant behavior, and they arrive at different conclusion. For example, Peng and Zeng (2016) find that workplace ostracism exerts no influence on deviant behavior without moderating role of 360 degree feedback, which is different from most previous studies (Shafique et al., 2021). Also, in the Chinese context, some researchers have proposed that high differential order climate and power distance may make Chinese employees think workplace ostracism is reasonable, so they are more likely to tolerate it without exhibiting negative attitudes or behaviors (Chen and Tu, 2017). Jiang and Zhang (2021) confirm that high collectivism value in Chinese organizations will enable the ostracized employees to obtain the high-performance rate *via* obedient behaviors. Therefore, we suspect that the different results about workplace ostracism brought forth by various studies are due to different mediating mechanisms, moderating effects, and theory perspectives. In light of this, to comprehensively examine the impact of workplace ostracism on employees’ behavior, the current research focuses on whether and how workplace ostracism leads to deviant workplace behavior on the part of employees. Pure emotional revenge or the loss of resources is not sufficient to fully explain the complex relationship between workplace exclusion and employee behavior, as there must be other key factors.

Self-determination theory (SDT), which was proposed by Deci and Ryan (2000), holds that individuals’ behavior is based on different types of work motivation (including autonomous motivation and control motivation). The most important way to promote work motivation is to make the external situation meet employees’ three basic psychological needs: autonomy, competence, and relatedness (Deci and Ryan, 2000). When these basic psychological needs are satisfied, an individual’s work motivations are enhanced, so the belief that one’s work is meaningful and one’s confidence in career development are increased (Shi et al., 2018; Luo et al., 2020); this, in turn, promotes positive behaviors (Guo and Cheng, 2021). In contrast, if basic psychological needs are unsatisfied, the meaningfulness of one’s work is reduced, stimulating negative work attitudes and behaviors, such as work disengagement (Li et al., 2020). Therefore, from the perspective of SDT, the first and important objective of the current research is to examine the mediating role of basic psychological needs between workplace ostracism and deviant behavior. Specifically, as a kind of “cold violence,” workplace ostracism brings about a negative external environment, which can reduce the fulfillment of basic psychological needs of employees and thus trigger their deviant behavior.

However, the extent of workplace ostracism’s influence on employees’ basic psychological needs varies with the different individual characteristics. In this regard, we introduce the perceived inclusive climate as a key contextual factor to moderate the relationship between workplace ostracism and basic psychological needs. Perceived inclusive climate is defined as shared perceptions of whether the organization treats everyone fairly, accepts or attaches importance to different opinions, and encourages everyone to be in a core decision-making position (Nishii, 2013), such as the perception of employment equity and diversity, respect for organizational cultural diversity, and accepting subordinates’ suggestions. In recent years, diversity in China’s labor force has increased markedly by including women, post-00s, returnees from overseas, new migrants, and gig economy workers. Therefore, inclusive management has become important for Chinese enterprises (Xu and Zhang, 2018). Employees with a higher perceived inclusive climate can better realize the recognition of their identity and value from the organization, which can alleviate the negative impact brought on by the differences (Nishii, 2013; Guillaume et al., 2014; Nelissen et al., 2017). Most studies about inclusive climate perception have been carried out in western contexts, so the second objective of our research is to position employees’ perception of organizational inclusion as a pivotal and underexplored moderator in Chinese organizations.

**FIGURE 1:**
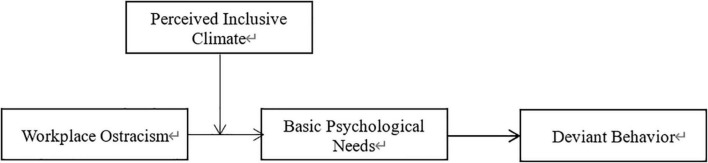
Proposed theoretical model.

This study makes the following theoretical contributions. First, we explore how and why workplace ostracism influences deviant behavior. Based on SDT, our mediating approach (i.e., the mediating role of basic psychological needs) contributes to the literature on workplace ostracism and deviant behavior by revealing how and why ostracism is a catalyst for deviant behavior. This novel approach can inform researchers and managers on how deviant behavior can be reduced. Second, we identify a contextual factor (perceived inclusive climate) as an important boundary condition when exploring the fluctuating effects of workplace ostracism in Chinese culture, thus contributing to the literature on workplace ostracism and culture. Our conclusion help researchers understand the negative effects of workplace ostracism from a contingency perspective. Third, the current study provides a more comprehensive view of how workplace ostracism affects employee behavior, including such aspects as employees’ behavior, psychological changes, and perceptions of organizational situations. Figure 1 depicts our theoretical model.

## Theory and hypotheses

### Workplace ostracism and deviant behavior

We argue that workplace ostracism triggers employees’ deviant behavior. Workplace deviant behavior refers to “voluntary behavior that violates significant organizational norms and in so doing threatens the wellbeing of an organization, its members, or both” (Robinson and Bennett, 1995, p. 556), which is historically considered negative deviant behavior (Bennett and Robinson, 2000). Workplace ostracism is a kind of workplace stressor that destroys normal interpersonal communication in the work environment (Downey and Feldman, 1996; Ferris et al., 2008; Matt et al., 2020); hence, employees may exhibit deviant behavior to relieve stress and conserve resources (Koeske et al., 1993; Cui et al., 2021). Through deviant behavior, employees can not only express their dissatisfaction with the organization but also release their own workplace pressure using self-protection. Additionally, as a kind of “cold violence,” workplace ostracism is an unfair interactive event in an organization, so employees may retaliate through negative reciprocity (Ferris et al., 2008), namely, by engaging in tit-for-tat behavior. Therefore, we propose the following hypothesis:

*Hypothesis 1:* Workplace ostracism is positively related to employee deviant behavior.

### Mediating role of basic psychological needs

According to SDT, basic psychological needs are satisfied or rejected under the influence of internal and external environments to stimulate individuals’ motivation or tendency and finally to promote certain behaviors (Deci and Ryan, 1985). As a negative external environment, workplace ostracism hinders employees’ basic psychological needs and induces deviant workplace behavior. This means basic psychological needs may play a mediating role between workplace ostracism and deviant behavior. Basic psychological needs include autonomy, competence, and relatedness needs (Deci and Ryan, 2000). If any of these three needs is frustrated or not satisfied, the individual’s mental health deteriorates, causing unhappiness. When employees feel that they are excluded or ignored by their colleagues or superiors, their psychological needs are unfulfilled, so their mental health declines, leading to negative emotions like stress, emotional exhaustion, and depression (Choi, 2020). Workplace ostracism manifests itself as refusal to work together, avoidance of conversation, and even ignoring the existence of others. It cuts off social contact between ostracized individuals and others. Through negation and neglect, workplace ostracism conveys implicit punishment information to organizations, that is, an ostracized employee feels unwelcome, insignificant, and disrespected, which hinders the individual from establishing an equal or trusted interpersonal relationship with others (Paşamehmetoğlu et al., 2022). Individuals always want to control their surroundings to reduce the impact of uncertainty. Ostracized employees cannot obtain responses from other colleagues, so they cannot interact normally according to their own needs. Additionally, they cannot cooperate with others, express ideas, and take actions, which may lower their willingness to work. Previous studies have shown that social ostracism negatively affects basic needs such as self-esteem, sense of belonging, control, and self-worth (Al-Atwi et al., 2021). Therefore, we can infer that as a special form of social ostracism in the work context, workplace ostracism has a significant negative effect on the basic psychological needs of employees.

The fulfillment of basic psychological needs can further restrain employees’ deviant behavior. As an essential, congenital nutrient of individual psychological growth, integration, and happiness (Deci and Ryan, 2000), basic psychological needs are important sources of self-motivation. According to SDT, individual behavior is driven by motivation, including extrinsic and intrinsic motivation, and in order to drive behavior, extrinsic motivation needs to be transformed into internal motivation. The satisfaction of basic psychological needs can promote individuals to develop in a positive and healthy direction by generating internal motivation and internalizing external motivation (Ryan and Deci, 2017; Thomas et al., 2018). Specifically, deviant behavior is the retaliation of employees against organization (because they are dissatisfied). Basic psychological needs can improve the internal motivation of individuals through willingness support, relationship support, and positive feedback, which help employees experience happiness. The satisfaction of autonomy, competence, and relatedness needs allows individuals to show their real strengths in the workplace, be competent in the performance of their duties, actively learn new knowledge and skills, and gain good interpersonal relationship. The satisfaction of these needs provides conditions for the improvement of individual work control and empathy so that employees will not choose to behave in a manner detrimental to their colleagues or the organization. Thus, it reduces the likelihood of workplace deviance. In addition, prior studies show that the fulfillment of basic psychological needs can positively predict mental health and good relationships (Patrick et al., 2007) and thereby encourages individuals to have a greater sense of life significance (Eakman, 2014). Additionally, individuals with higher satisfaction of basic psychological needs can show fewer symptoms of depression and less indifferent behavior (Ferrand et al., 2015).

From the perspective of SDT, workplace ostracism is a negative external factor. This kind of “cold violence” frustrates the basic psychological needs of employees and hence cannot inhibit the occurrence of deviant workplace behavior. Therefore, we propose the following hypothesis:

*Hypothesis 2:* Basic psychological needs mediate the positive relationship between workplace ostracism and deviant behavior.

### Moderating role of perceived inclusive climate

As noted previously, the negative effects of workplace ostracism on basic psychological needs may depend on the level of perceived inclusive climate, which reflects the individual’s perceived position in the organization or interpersonal network (Schein, 1971). Existing studies have found that an organization’s tolerance of diversity can encourage employees to improve and apply their own capabilities, thus selflessly contributing to the organization (Shore et al., 2011). The higher the perceived organizational inclusion of employees, the easier it is to reduce their deviant behavior (Blau and Andersson, 2005). Similar to the perception of organizational fairness, organizational inclusive climate perception is a positive factor felt by employees about organizations, so it may inhibit negative influences, like any other positive factor.

Organizations with high inclusive climate can build a working environment that is able to accommodate different individuals, integrate the diversity of employees, and accept different opinions, values, and behaviors. When the organization’s rules are highly consistent with the individual’s psychological need for inclusiveness and respect, employees perceive organizational inclusion positively, which includes respect for individual uniqueness, a high sense of organizational justice, diversity of cultural compatibility, and willingness to listen to employees’ input. Thus, the perception of being ostracized can be balanced by an inclusive climate so as to inhibit its negative influence on basic psychological needs.

In contrast, organizations with low inclusive climate may be seen as guilty of unfairness in the promotion process, performance evaluation, and income distribution, such that employees are unable to express their ideas and advice through normal channels, and may even feel that their dignity and self-worth have been disregarded. Thus, ostracized individuals cannot relieve stress and cope with emotions; they are more easily frustrated in their basic psychological needs in organizations with low inclusiveness. Therefore, we propose the following hypothesis:

*Hypothesis 3:* The perceived inclusive climate moderates the relationship between workplace ostracism and basic psychological needs, such that the negative relationship is weaker for employees with a high perceived climate of inclusion than for those with a low perceived climate of inclusion.

## Materials and methods

### Participants and procedure

To ensure the availability of data (Zhao et al., 2021), and considering that this research topic is not limited to specific industries and regions, the data were acquired from dozens of enterprises in China through the researchers’ acquaintance circles. Before the data collection, participants were told that the survey would only be used for academic research and would not infringe on their privacy. All participants completed the survey on a voluntary basis. Depending on the participants’ willingness and convenience, data were collected through web-based surveys and offline questionnaires. A total of 330 questionnaires were sent out and 313 were returned, with an effective recovery rate of 94.8%. After excluding 66 invalid questionnaires, the final dataset comprised 247 responses (92 offline and 155 web-based), constituting a final effective response rate of 79%. The demographics of the final sample were as follows: 45.3% male, 59.1% unmarried, most respondents’ age (72.1%) was under 30 years, 90.7% of respondents had earned a bachelor’s degree or above, and most (72.9%) respondents’ tenure in their company was over 2 years. To ensure there was no significant difference between offline and web-based surveys, we conducted an independent sample *t*-test at first, which confirmed that there was no significant difference (the *p*-values of all variables are more than 0.05). The results are shown in Table 1.

**TABLE 1:** *T*-test of offline and web-based surveys.

Variable	Offline (*n* = 92)	Web-based (*n* = 155)	*t*-value	*p*
1. Gender	0.43 ± 0.50	0.46 ± 0.50	0.45	0.65
2. Marital status	1.38 ± 0.49	1.45 ± 0.54	0.95	0.34
3. Age	1.28 ± 0.56	1.34 ± 0.56	0.72	0.40
4. Education	2.98 ± 0.36	3.01 ± 0.46	0.50	0.617
5. Tenure	2.16 ± 1.07	2.41 ± 1.01	1.80	0.07
6. Workplace ostracism	1.61 ± 30.45	1.69 ± 0.56	1.28	0.20
7. Basic psychological needs	3.28 ± 0.20	3.25 ± 0.28	−0.99	0.32
8. Deviant behavior	1.61 ± 0.51	1.61 ± 0.48	−0.12	0.91
9. Perceived climate for inclusion	3.41 ± 0.53	3.32 ± 0.58	−1.21	0.23

*N* = 247. For gender, 1 = male, 0 = female. For marital status, 1 = unmarried, 2 = married, and 3 = divorced. For age (in years), 1 = 30 or under, 2 = 31–40, 3 = 41–50, and 4 = over 50. For education, 1 = some high school, 2 = high school degree, 3 = bachelor’s degree, and 4 = graduate degree. For tenure, 1 = less than 2 years, 2 = 2–5 years, 3 = 5–10 years, and 4 = more than 10 years. Coefficient alphas are reported along the diagonal in parentheses. *p < 0.05, **p < 0.01.

### Measures

To gather the data, we adopted established scales available in the open domain. Unless otherwise stated, all ratings were on a 5-point Likert scale (from 1 = *Strongly disagree* to 5 = *Strongly agree*). All scale items underwent a translation and back translation process (Brislin, 1986) to ensure the internal validity of the translated scales.

#### Workplace ostracism

We measured workplace ostracism using ten items (*a* = 0.91) developed by Ferris et al. (2008). Sample items are “Others left the area when you entered,” “You noticed others would not look at you at work,” and “Others refused to talk to you at work.” The values of confirmatory factor analysis (CFA) of the scale were [χ^2^ (31) = 73.31, χ^2^/df = 2.37, *p* < 0.001, CFI = 0.973, TLI = 0.961, RMSEA = 0.074].

#### Basic psychological needs

We measured basic psychological needs using a three-dimensional scale developed by Gagné and Deci (2005), with a total of 21 items (*a* = 0.77), including competency needs (8 items), relationship needs (6 items), and autonomous needs (7 items). Sample items include “I feel like I can decide on my own how to live my life” (autonomy), “I truly like the people I interact with” (relatedness), and “I often do not feel very capable” (competence, reversed). The CFA values of the scale were [χ^2^ (170) = 218.77, χ^2^/df = 1.29, *p* < 0.001, CFI = 0.936, TLI = 0.921, RMSEA = 0.034].

#### Deviant behavior

We measured deviant behavior using a two-dimensional scale developed by Bennett and Robinson (2000), with a total of 19 items (*a* = 0.92), including interpersonal deviance (7 items), and organizational deviance (12 items). Sample items include “I played a mean prank on someone at work” and “I come in late to work without permission.” The CFA values of the scale were [χ^2^ (130) = 270.07, χ^2^/df = 2.08, *p* < 0.001, CFI = 0.935, TLI = 0.915, RMSEA = 0.066].

#### Perceived inclusive climate

We measured perceived inclusive climate using 15 items (*a* = 0.86) shortened by Nishii (2013). The scale is divided into three dimensions: fairly implemented employment practices (5 items), such as “The performance review process is fair in this unit”; integration of differences (6 items), such as “This unit commits resources to ensuring that employees are able to resolve conflicts effectively”; and inclusion in decision-making (4 items), such as “In this unit, everyone’s ideas for how to do things better are given serious consideration.” The CFA values of the scale were [*a* = 0.86, χ^2^ (84) = 156.03, χ^2^/df = 1.86, *p* < 0.001, CFI = 0.932, TLI = 0.915, RMSEA = 0.059].

#### Control variables

We controlled for several demographic variables that might have an impact on employees’ deviant behavior, including gender, marital status, age, education, and tenure. Martinko et al. (2002) find that male employees are more likely to engage in transgressive behavior when they are ostracized in the workplace. Hollinger and Clark (1983) find that employees with lower social status or lower levels of education have lower loyalty to the organization, and they are more likely to engage in transgressive behaviors to release negative emotions.

### Analytical strategy

**FIGURE 2:**
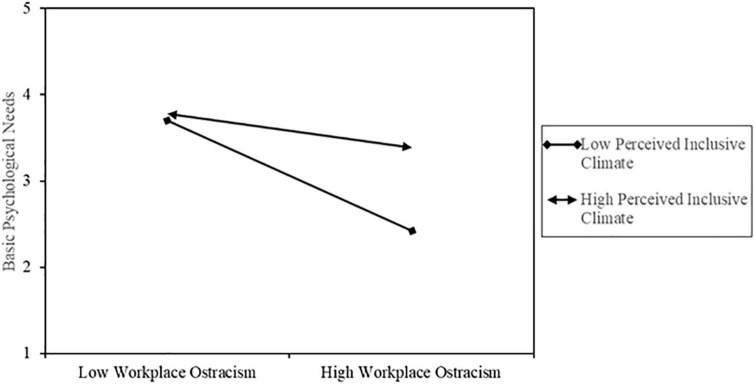
The interactive effect between workplace ostracism and perceived climate for inclusion on basic psychological needs.

A *t*-test was conducted first to ensure that there was no significant difference between two groups from offline and web-based surveys (see Table 1). We then employed the Harman single-factor method (Harman, 1976) to demonstrate that common-method variance was not a serious problem in this study. Thereafter, a series of confirmatory factor analyses were conducted to confirm the dimensionality and discriminant validity of our multi-item measures. Furthermore, we added an unmeasured common method factor with all measures as indicators and set the method factor to be uncorrelated with the other latent variables (see Table 2). With descriptive statistics, we provide preliminary evidence for subsequent hypothesis testing (see Table 3). To test hypothesis H1 and preliminarily examine the mediating effect of basic psychological needs as well as the moderating effect of the perceived inclusive climate, we did hierarchical regression analysis with SPSS 23. The results are presented in Table 4. Next, to assess the size of indirect effects stipulated in the hypotheses, we adopted a bootstrapping strategy using Mplus 8.3 because this method does not assume the shape of the sampling distribution and offers greater statistical power and more accurate estimation than conventional methods. Finally, we conducted a simple slope test and drew a diagram of the moderating role of the perceived inclusive climate in the relationship between workplace ostracism and basic psychological needs in Figure 2.

**TABLE 2:** Results of confirmatory factor analyses.

Model	χ^2^ (*df*)	Δχ^2^ (Δ *df*)	RMSEA	SRMR	CFI
WO; BPN; DB; PCI	316.46 (129)		0.08	0.05	0.91
WO; BPN + DB; PCI	381.36 (132)	64.90 (3)**	0.09	0.07	0.88
WO; BPN + PCI; DB	372.74 (132)	56.28 (3)**	0.09	0.07	0.88
WO + PCI; BPN + DB	567.79 (134)	251.33 (5)**	0.11	0.10	0.79
WO + BPN + DB + PCI	669.12 (135)	352.67 (6)**	0.13	0.10	0.74
WO; BPN; DB; PCI; CM	313.88 (128)	−2.58 (1)	0.08	0.05	0.91

*N* = 247. RMSEA, root-mean-square error of approximation; SRMR, standardized root-mean-square residual; CFI, comparative fit index; CM, common method; WO, workplace ostracism; BPN, basic psychological needs; PCI, perceived climate for inclusion. **p < 0.01.

**TABLE 3:** Means, standard deviations, and correlations of the focal variables.

Variable	Mean	*SD*	AVE	CR	1	2	3	4	5	6	7	8	9
1. Gender	0.45	0.50	−	−	−								
2. Marital status	1.42	0.52	−	−	0.12	−							
3. Age	1.32	0.56	−	−	0.13*	0.53**	−						
4. Education	3.00	0.43	−	−	–0.07	0.01	–0.05	−					
5. Tenure	2.32	1.04	−	−	0.16*	0.63**	0.68**	−0.14*	−				
6. Workplace ostracism	1.67	0.52	0.54	0.92	0.01	–0.02	0.03	0.04	0.05	(0.91)			
7. Basic psychological needs	3.54	0.37	0.57	0.69	–0.03	0.16*	0.15*	0.08	0.11	−0.39**	(0.77)		
8. Deviant behavior	1.61	0.49	0.69	0.81	0.15*	0.03	0.01	–0.03	0.15*	0.32**	−0.27**	(0.92)	
9. Perceived climate for inclusion	3.36	0.56	0.52	0.76	0.05	–0.09	–0.04	–0.01	–0.12	0.05	0.20**	–0.05	(0.86)

*N* = 247. *SD*, standard deviation. For gender, 1 = male, 0 = female. For marital status, 1 = unmarried, 2 = married, and 3 = divorced. For age (in years), 1 = 30 or under, 2 = 31–40, 3 = 41–50, and 4 = over 50. For education, 1 = some high school, 2 = high school degree, 3 = bachelor’s degree, and 4 = graduate degree. For tenure, 1 = less than 2 years, 2 = 2–5 years, 3 = 5–10 years, and 4 = more than 10 years. Coefficient alphas are reported along the diagonal in parentheses. *p < 0.05, **p < 0.01.

**TABLE 4:** Regression results.

Variables	Basic psychological needs				Deviant behavior			
	Model 1	Model 2	Model 3	Model 4	Model 5	Model 6	Model 7	Model 8
Gender	–0.04	–0.04	–0.05	–0.04	0.13*	0.13*	0.12*	0.12*
Marital status	0.08	0.05	0.06	0.05	–0.07	–0.04	–0.04	–0.03
Age	0.07	0.07	0.05	0.05	–0.13	–0.14	–0.11	–0.12
Education	0.07	0.09	0.09	0.10	0.01	–0.01	0.03	0.01
Tenure	–0.00	–0.03	0.03	0.03	0.13*	0.11*	0.13**	0.11**
Workplace ostracism (WO)		−0.28**	−0.29**	−0.31**		0.30**		0.24*
Basic psychological needs							−0.37**	−0.23*
Perceived climate for inclusion (PCI)			0.16**	0.17**				
WO × PCI				−0.16*				
*R* ^2^	0.40	0.19**	0.25**	0.27*	0.05*	0.15**	0.12**	0.17*
Δ*R*^2^	0.40**	0.15**	0.06**	0.02*	0.05*	0.10**	0.07**	0.02*
*F*	1.99	9.59**	11.59**	10.91**	2.66*	7.09**	5.60**	7.19**

*N* = 247. *p < 0.05, **p < 0.01.

## Results

### Common method variance test

There might be common method variance given that all the data came from respondents’ self-evaluation. Therefore, we employed the Harman single-factor method (Harman, 1976) to test whether this variance exists. Sixty-five items from exploratory factor analysis (EFA) were utilized in the test. The results show that 17 factors’ eigenvalues exceed 1, which explains 68.40% of the total variance. The first factor referring to autonomy explains 16.13%, which is significantly lower than 50%. These results clearly demonstrate that common method variance was not a serious problem in this study (Podsakoff et al., 2003; Malhotra et al., 2006).

### Validity test

Prior to hypothesis testing, we conducted a set of confirmatory factor analyses using Mplus software to ensure that the four-factor model (workplace ostracism, basic psychological needs, deviant behavior, and perceived climate for inclusion) had satisfactory discriminant validity. The CFA results presented in Table 2 show that the hypothesized four-factor model (the four variables as separate factors) reached a reasonable level of fit [χ^2^ (129) = 316.46, RMSEA = 0.08, SRMR = 0.05, CFI = 0.91]. Furthermore, as shown in Table 2, the chi-square difference test illustrates that the four-factor model yielded a significantly better fit than the three-factor models (with three of the four variables combined as a factor), the two-factor model, and the single-factor model (with the four variables as one combined factor). These results provided construct validity evidence of the four latent variables in the Chinese context.

Furthermore, we included unmeasured common method factors in the hypothesized measurement models to examine whether the method effect was present. As shown in Table 2, the measurement model including the four factors and a CM (common method) factor also fit the data well; however, it did not significantly improve the model fit [Δχ^2^ (Δ*df* = 1) = −2.58, *p* > 0.05]. It further certifies that common-method variance was not a serious problem in our study.

In addition, data for the four latent constructs (workplace ostracism, basic psychological needs, deviant behavior, and perceived climate for inclusion) were analyzed for composite reliability (CR) and convergent validity. As indicated in Table 3, the values of CR for all four constructs were in the range 0.69–0.92, which suggests that the constructs of the study have excellent internal consistency. Convergent validity of the constructs was confirmed through average variance extracted (AVE). As shown in Table 3, the values of AVE for all four constructs were above 0.5, which shows that convergent validity was not a problem in the study.

### Descriptive statistics

Reported in Table 3 are the descriptive statistics and correlations among the focal variables. The results suggest a negative correlation between workplace ostracism and basic psychological needs (*r* = −0.39, *p* < 0.01), a positive correlation between workplace ostracism and deviant behavior (*r* = 0.32, *p* < 0.01), and a negative correlation between basic psychological needs and deviant behavior (*r* = −0.27, *p* < 0.01), which provides preliminary evidence for subsequent hypothesis testing.

### Hypothesis tests

We tested the model *via* hierarchical regression using SPSS. The results are shown in Table 4.

Hypothesis 1 proposes that workplace ostracism is positively associated with deviant behavior. As anticipated, it receives support (*B* = −0.30, *p* < 0.01, Model 6).

Hypothesis 2 proposes that workplace ostracism exerts an indirect effect on deviant behavior *via* basic psychological needs. To compare Model 7 and Model 8, we add workplace ostracism in Model 8 based on Model 7, which still suggests a negative association between basic psychological needs and deviant behavior (*B* = −0.23, *p* < 0.05, Model 7). Although the effect is weaker, it is still significant. Furthermore, to compare Model 6 and Model 8, we embed basic psychological needs in Model 8 based on Model 6, which also reveals a positive effect of workplace ostracism on deviant behavior (*B* = 0.24, *p* < 0.05, Model 8). To sum up, it can be concluded that workplace ostracism indirectly affects employees’ deviant behavior by affecting their basic psychological needs. Specifically, workplace ostracism reduces the fulfillment of employees’ basic psychological needs, which increases employees’ deviant behavior, with its mediating role partially significant. Combined, this indirect effect as Hypothesis 2 is pronounced.

To further verify Hypothesis 2, we followed the bootstrapping-based analytic approach of Edwards and Lambert (2007). Based on 5,000 resamples, our Mplus output shows that the indirect effect of workplace ostracism on deviant behavior through basic psychological needs is significant (*B* = 0.06, *p* < 0.05, *SE* = 0.03, 95% CI = 0.01–0.11, excluding zero), lending support to Hypothesis 2.

With respect to the interaction hypothesis (Hypothesis 3), the results in Table 4 demonstrate that after including the control variables and main effects of workplace ostracism, perceived climate for inclusion had a negative moderating effect on the relationship between workplace ostracism and basic psychological needs (*B* = −0.16, *p* < 0.05, Model 4).

We further elucidate the pattern of the moderating effect of perceived climate for inclusion by drawing the interaction effect graph (Dodhia, 2005) depicted in Figure 2, which shows that the nature of the interaction was consistent with our expectations, such that workplace ostracism is more positively related to basic psychological needs when the perceived climate for inclusion is lower (*B* = −0.40, *p* < 0.01) than when it is higher (*B* = −0.22, *p* < 0.01). Thus, Hypothesis 3 is supported.

## Discussion

Based on SDT, this study examines and tests how workplace ostracism triggers deviant behavior in employees. We find that as a form of “cold violence,” workplace ostracism has a significant effect on employees’ deviant behavior. Workplace ostracism influences deviant behavior indirectly through basic psychological needs; that is, employees’ basic psychological needs mediate the positive relationship between workplace ostracism and deviant behavior. Perceived inclusive climate moderates the negative relationship between workplace ostracism and basic psychological needs, such that the relationship is weaker when organizational inclusion as perceived by employees is high than when it is low.

### Theoretical implications

This study makes several important theoretical contributions. First, drawing on SDT (Deci and Ryan, 2000), we provide a novel perspective on the negative effects of workplace ostracism. Previous studies applied COR or reciprocity theory to explore the negative behavior caused by workplace ostracism. According to negative reciprocity theory, workplace ostracism shows others’ disregard, indifference, and exclusion, and individuals retaliate in the same way, such as by reducing positive out-of-role behaviors (Hitlan et al., 2006). However, this perspective ignores the impact of workplace ostracism on the deeper intrinsic motivation of individuals. If an ostracized worker’s basic psychological needs are frustrated or not satisfied, their external motivation isn’t transformed into internal motivation through workplace ostracism, resulting in certain negative behaviors, which confirms and extends the conclusion of previous research (Li et al., 2020).

Second, through SDT this study identifies and investigates an important new pathway by explaining how basic psychological needs [the key branch of SDT (Wu et al., 2018)] mediate the relationship between workplace ostracism and deviant behavior. Workplace ostracism is a negative external factor. It frustrates the basic psychological needs of employees (Williams, 2009), causing the occurrence of deviant workplace behavior. As a result, our study can help researchers understand how workplace ostracism can lead to employees’ deviant behavior. Additionally, it contributes to the literature on basic psychological needs by exploring their mediating effect in response to Bedi’s (2021) call for research on “the specific process of demand strengthening affecting counterproductive work behavior.”

Finally, our study enlightens the workplace ostracism literature by investigating perceived inclusive climate as a moderating factor, qualifying the association between workplace ostracism and basic psychological needs through SDT. In particular, we know that the negative results of workplace ostracism need boundary conditions; that is, for employees with a high perception of organizational inclusion, there is a lower negative effect of workplace ostracism on basic psychological needs than for employees with a low perception of organizational inclusion. This confirms the research of Dwertmann and Boehm (2016) and Li et al. (2017): a climate with high inclusiveness increases the positive effect of antecedents and mediators and reduces their negative effect. These findings can help researchers understand the effect of workplace ostracism, that is, how workplace ostracism generates passive results for different employees with different perceptions of inclusive climate. At the same time, the findings supply theoretical insight into how to avoid the negative effect of workplace ostracism. Besides, we confirm the positive effect of the perceived inclusive climate in the Chinese context, which enriches the cultural ramifications of this western concept (Xu and Zhang, 2018). Maybe, it’s response to the review of Bedi (2021), too, which said organizations can benefit from promoting an inclusive culture to avoid the negative of workplace ostracism.

### Practical implications

In practice, deviant workplace behavior often leads to damage to the interests of organizations, which, in turn, seriously affects their operations and development (Camara and Schneider, 1994; Robinson and Bennett, 1995). By examining the relationship between workplace ostracism and deviant behavior, this study guides organizations in how to reduce such behavior.

First, enterprises should find ways to alleviate the pressure of certain interpersonal dynamics, such as workplace ostracism, so as to help reduce employees’ deviant behavior that may cause damage to the organization. For example, with the development of information technology, online and remote work can reduce interpersonal pressure and tension (Raghuram and Fang, 2014; Chen and Tu, 2017).

Furthermore, managers should encourage employees to acquire positive energy in work and life, improve the satisfaction of basic psychological needs, and reduce employees’ deviant behaviors. Deci and Ryan (2000) believe that people are naturally attracted to activities that may improve their competence, build relatedness with social groups, and experience autonomy. When an environment meets an individual’s three basic psychological needs, that individual can develop positively and healthily. Existing studies have confirmed that work needs and work resources can affect the satisfaction of an individual’s basic psychological needs in the workplace. Specifically, the more demanding the work, the lower the satisfaction of people’s basic psychological needs, while the more resources the work, the higher the fulfillment of people’s basic psychological needs (Wu et al., 2018). Therefore, organizations should provide opportunities for employees to learn new knowledge and skills continuously through work and practice, build communication platforms to facilitate employee interaction, and foster harmonious organizational cultures to increase employees’ interpersonal resources and eventually satisfy their basic psychological needs.

Finally, an inclusive organizational culture should be established to promote harmonious coexistence between leaders and subordinates. Managers should realize the significance of employees’ inclusive climate perception for the satisfaction of their basic psychological needs. Therefore, they should make rules and organize some activities to build an inclusive organizational culture. For managers, the ability to build an inclusive organizational culture is vital (Acquavita et al., 2009). Researchers have confirmed that transformational leadership, servant leadership, moral leadership, and spiritual leadership have positive effects on teams’ inclusive culture (Gotsis and Grimani, 2016), so managers can show their related traits to strengthen the inclusive organizational climate. For organizations, learning and integration can form a strong inclusive culture; that is, organizations can rely on members’ different backgrounds to achieve strategic organizational goals (Ely and Thomas, 2001), so organizations should integrate employees and their cultural differences to support this. In addition, human resource management practices (i.e., hiring diverse employees) can promote an inclusive organizational culture (Shore et al., 2009), for example, by treating diverse employees fairly and encouraging employees to participate in decision-making.

### Limitations and future research

Like all studies, our work is subject to several limitations, which provides exciting areas for potential future research. First, the data came from a single source and were self-reported, which may have inflated the correlations and thus increased the risk of common method bias (Podsakoff et al., 2003). As noted, we apply the Harman single-factor method and unmeasured common method factors to indicate that common method variance was not a serious issue, but the concern of common method variance could be further mitigated by refining the research design, for example, by collecting multisource data and conducting longitudinal research. At the same time, future scholars are strongly encouraged to carry out a scenario-based experiment to manipulate workplace ostracism within our research framework to test and further probe its effect (Jiang and Zhang, 2021).

Second, our study is limited to Chinese employees and thus has cultural limitations. For example, Chinese society is characterized by collectivism, emphasizing obligation and loyalty to the group (Grossmann and Na, 2014). Even when perceiving workplace ostracism, a highly collective culture makes employees pay more attention to the benefits of organizations, leading to fewer negative reactions. However, culture as a “face concept” may reinforce the negative effects of workplace ostracism. Face, as the social value of individual communication, represents a kind of identification and respect (Goffman, 1955). Workplace ostracism expresses exclusion and disregard in the workplace. Ostracized individuals with strong face values perceive a stronger threat of “losing face,” resulting in more emotional and behavioral changes, but perhaps also to reintegrate in the organization more positively. Therefore, the effect of workplace ostracism can be different because of collective and face view in traditional Chinese culture. In future studies, this problem could be avoided by conducting cross-cultural research.

Third, we collected data without considering different industries, different kinds of organizations (such as MNCs and SMEs), and this may have influenced the results. For example, there are more and more studies on the influence of workplace ostracism in the services industry (Paşamehmetoğlu et al., 2022). That’s to say industry may influence the result, which is ignored by our study. Perhaps future research on this topic can consider different industries, types of organization, and organizational cultures.

Fourth, this study investigated the mediating role of basic psychological needs in the effect of workplace ostracism on deviant behavior from the perspective of SDT, yet there may be other underlying mediating mechanisms that are plausible. For example, one could explore the mediating effect of an individual’s sense of prediction and control on workplace ostracism to self-conception and behavior from the lens of self-verification theory.

Finally, this study framed and examined perceived inclusive climate as a potentially crucial moderating factor or boundary condition qualifying the linkage between workplace ostracism and basic psychological needs, yet alternative variables could be explored in the future, such as generational differences. Specifically, there may be significant differences in thinking patterns and cognitive styles between different generational groups. Younger generations have a greater sense of entitlement, value accountability, and challenge authority (Laird et al., 2015), and may therefore react more strongly and be more sensitive to perceived workplace ostracism. In addition, we tested the moderating mediated role of perceived inclusive climate, but the influence is not significance. Scholars should explore the reasons about this by collecting data from different samples, adopting different methods and so forth.

## Data availability statement

The datasets presented in this article are not readily available because the datasets cannot be used to commercial application. Requests to access the datasets should be directed to JL.

## Ethics statement

The studies involving human participants were reviewed and approved by School of Business Administration, Southwestern University of Finance and Economics. The participants provided their written informed consent to participate in this study.

## Author contributions

All authors made substantial contributions to conception and design, acquisition of data, or analysis and interpretation of data; took part in drafting the manuscript or revising it critically for important intellectual content; agreed to submit to the current journal; gave final approval of the version to be published; and agreed to be accountable for all aspects of the work.
